# Validation of Drug-Like Inhibitors against *Mycobacterium Tuberculosis* L-Aspartate α-Decarboxylase Using Nuclear Magnetic Resonance (^1^H NMR)

**DOI:** 10.1371/journal.pone.0045947

**Published:** 2012-09-28

**Authors:** Reetu Sharma, Mara Florea, Werner M. Nau, Kunchithapadam Swaminathan

**Affiliations:** 1 Department of Biological Sciences, National University of Singapore, Singapore, Singapore; 2 School of Engineering and Science, Jacobs University, Bremen, Germany; Concordia University Wisconsin, United States of America

## Abstract

The catalytic activity of L-aspartate α-decarboxylase (ADC) is essential for the growth of several micro-organisms, including *Mycobacterium tuberculosis* (Mtb), and has triggered efforts for the development of pharmaceutically active compounds against tuberculosis. The present study is a continuation of our recent chemoinformatics-based design approach for identifying potential drug-like inhibitors against MtbADC. We report an NMR-based protocol that allows label-free and direct monitoring of enzymatic conversion, which we have combined with a systematic testing of reported and newly identified potential inhibitors against MtbADC. Quantification of enzymatic conversion in the absence and presence of inhibitors allowed for a relative measure of the inhibitory effect (*k*_rel_). Among the newly identified compounds, D-tartrate, L-tartrate, and 2,4-dihydroxypyrimidine-5-carboxylate were found to inhibit the enzyme with *k*_rel_ values of 0.36, 0.38, and 0.54, respectively. In addition to the identification of potential building blocks for the development of therapeutic agents, the current study highlights the importance of electrostatic interactions governing enzyme-inhibitor binding.

## Introduction

L-aspartate α-decarboxylase (ADC, EC 4.1.1.11), encoded by the *E. coli panD* gene, is an enzyme responsible for the conversion of L-aspartate to β-alanine and its activity has been shown to be crucial for the growth of several microorganisms, including *Mycobacterium tuberculosis* (Mtb) [1]–[3]. In short, formation of β-alanine allows the synthesis of panthotenate (vitamin B_5_), the precursor of coenzyme A (CoA), which in turn plays an essential role in the metabolism and biosynthesis of fatty acids. The complex lipidoglycans found in the Mtb cell wall are therefore crucial for the intracellular replication, persistence, and virulence of the bacterium [4]–[6]. Impairing the pantothenate pathway by suppressing β-alanine formation has been shown to result in a significant decline in Mtb virulence [7], [8], which has motivated us to consider ADC as a potential target for therapeutic intervention against tuberculosis.

Recently, we have engaged in a chemoinformatics-based approach to identify potential drug-like inhibitors by structure-based virtual screening, which led to 28 lead molecules [9]. The purpose of our present investigation is to experimentally test a representative subset of the identified targets and contrast them with several compounds, which in part have been previously shown to be active in inhibition experiments [8], [10], [11]. Existing assays for ADC involve derivatization and separation steps [12], [13], radioactive labeling [1], [10] and laborious manometric quantification of the carbon dioxide released as a reaction by-product [14]. Extending our ongoing efforts in the design of enzyme assays [15], [16], including decarboxylase assays [17], we report a novel but simple ^1^H NMR protocol for monitoring ADC activity, which not only allows for direct structural information on the enzymatic reaction to be obtained and progress curves to be taken, but also serves as a convenient tool for inhibitor screening.

## Materials and Methods

### Inhibitors

**Figure 1:**
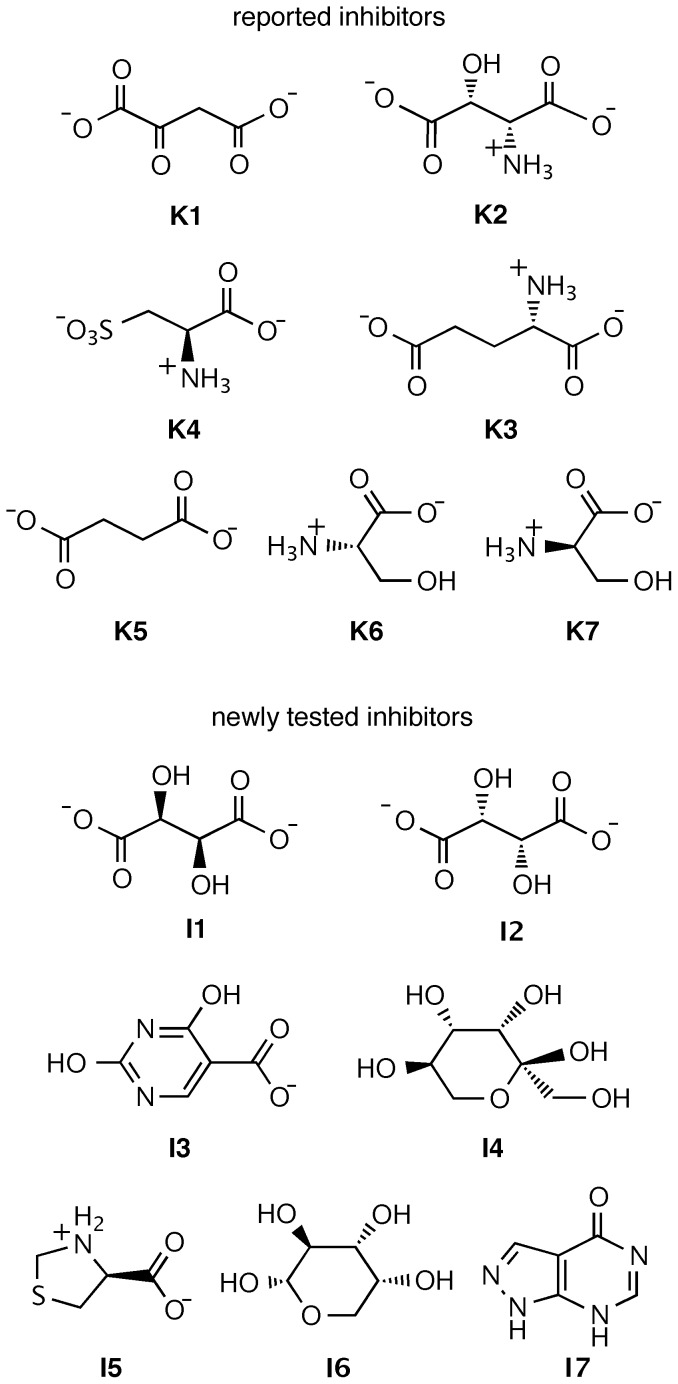
Chemical structures of known inhibitors against ADC (*K1–K7*) and computationally identified potential inhibitors obtained *via* virtual screening (*I1–I7*).

The previously reported and newly identified compounds that were tested in the present study for inhibitory effect against ADC are shown in Fig. 1. Oxaloacetate (***K1***), DL-*threo*-β-hydroxyaspartate (***K2***), L-glutamate (***K3***), L-cysteic acid (***K4***), succinate (***K5***), L-serine (***K6***), and D-serine (***K7***) had been tested previously, while D-tartrate (***I1***, ZINC00895296), L-tartrate (***I2***, ZINC00895301), 2,4-dihydroxypyrimidine-5-carboxylate (***I3***, ZINC00901606), D-tagatose (***I4***, ZINC03830878), (4S)-1,3-thiazolidin-3-ium-4-carboxylate (***I5***, ZINC00967474), α-D-arabinopyranose (***I6***, ZINC03606295), and 1,2-dihydropyrazolo[3,4-d]pyrimidin-4-one (***I7***, ZINC05177572) were tested for the first time, stimulated by the hits recently identified in the chemoinformatics study [9]. The compounds were purchased from Sigma-Aldrich, Germany (***K1–K7*** and ***I1–I4***), or from Labotest KG, Germany (***I5–I7***), in the highest commercially available purity (>97%).

### Protein preparation

*Mycobacterium tuberculosis* L-aspartate α-decarboxylase (MtbADC) was overexpressed with a C-terminal 6xHis tag in *E. coli* and purified as the cleaved tetrameric form by Ni^2+-^NTA affinity and gel filtration chromatography as described previously [2], [6]. The protein was further dialyzed against 10 mM Tris, pH 7.5.

### Enzyme assays

The enzymatic reactions were carried out in D_2_O (Sigma-Aldrich) using 1 mM L-aspartic acid as substrate in the absence or presence of 1 mM inhibitor. The reactions were initiated by addition of enzyme (2.83 µM, as determined by using an extinction coefficient ε_280_ = 6400 M^−1^cm^−1^ [2] and measured on a Varian Cary 4000 UV-Vis spectrophotometer. The enzymatic activity was followed by ^1^H NMR using a Jeol JNM-ECX 400 spectrometer.

## Results and Discussion

### Enzyme assays

**Figure 2:**
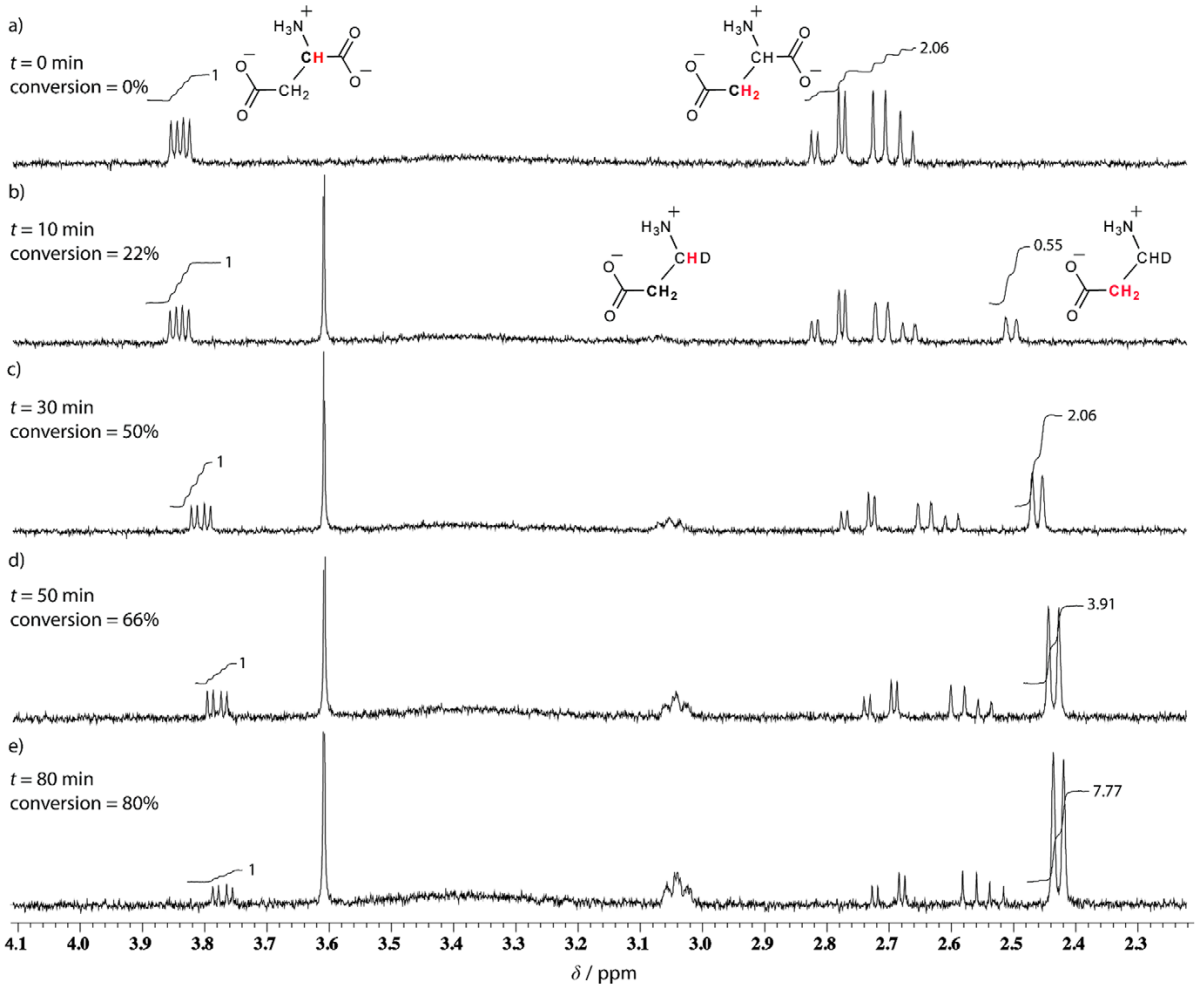
Selected ^1^H NMR spectra of 1 mM L-aspartate a) before and b)–e) 10–80 min after addition of 3 µM ADC in D_2_O at 25°C. The diminishing signals of L-aspartate and the emerging ones of those corresponding to β-alanine permitted a direct monitoring of the enzymatic transformation and integration of the proton signals allowed for a kinetic profiling of the reaction (*cf.* Fig. 3).

Before proceeding to inhibitor screening, a convenient assay for monitoring the ADC activity was established. Since during an enzymatic transformation, structural changes, which may be detected by differences in spectra of the substrate and product of the reaction, occur almost inevitably [18], [19], we opted for NMR spectroscopy as the technique of choice. Therefore, we validated a simple protocol using ^1^H NMR for monitoring the enzymatic depletion of L-aspartate and concomitant formation of β-alanine. The advantage of ^1^H NMR is that the technique is label-free and allows direct monitoring. To this end, the conversion of 1 mM L-aspartate in the presence of 3 µM ADC could be conveniently followed as shown in Fig. 2. L-aspartate shows two resonances, in a 1∶2 ratio (Fig. 2a). Upon addition of ADC, the signals corresponding to the product start to emerge and intensify with time while those of the substrate L-aspartate diminish and eventually completely disappear (Fig. 2 b–e). Note that, based on its structure, one would also expect two different ^1^H NMR peaks for β-alanine (this was confirmed by taking a spectrum for the commercial analyte). However, only the signals corresponding to the protons adjacent to the carboxylate group could be quantified in the course of the enzymatic reaction (at approximately δ = 2.44 ppm). This is a consequence of the fact that the reaction is carried out in D_2_O, and, therefore, the newly acquired β-alanine hydrogen is, in fact, a deuterium atom. This is also in line with both the broadness of the signal identified at approximately δ = 3.04 ppm and the splitting pattern (a doublet) of the upfield-shifted protons.The assay was optimized with respect to substrate and enzyme concentrations, such that the conversion rate was directly proportional to the enzyme concentration.

**Figure 3:**
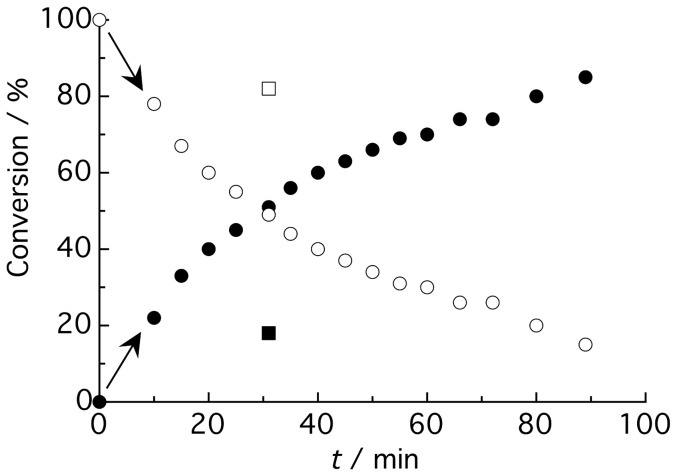
Kinetic monitoring of ADC activity carried out using 1 mM L-aspartate and 3 µM enzyme. The different points correspond to conversion percentages of the individual ^1^H NMR spectra taken at increasing reaction times after initiation of the reaction in D_2_O at 25°C. Percentage of product formation and substrate depletion is represented by filled and empty circles, respectively. The percentage of product and substrate after 30 min of the reaction in the presence of D-tartrate is represented by filled and empty squares, respectively.

Accordingly, the depletion of the L-aspartate substrate and the concomitant formation of β-alanine could be readily detected and, upon integration, also accurately quantified. This has finally allowed us to extract a kinetic profile of the enzyme in the absence of inhibitor (empty and filled circles in Fig. 3), which does not only depict a time-resolved monitoring of β-alanine formation as a result of the enzymatic activity, but also serves as a reference for subsequent inhibition studies (for instance, D-tartrate, empty and filled squares in Fig. 3). Since it is desirable to work in the linear region of the enzyme kinetic trace, we have used in our study 50% conversion in the absence of inhibitor as a reference point, which was achieved at *t* = 30–40 min, depending on the enzyme batch used.

### Inhibitors selected

Despite the relevance of ADC as a drug target, only a relatively small number of compounds have been reported to inhibit its activity [10]. Among these previously identified inhibitors, we have selected seven (***K1–K7***, Fig. 1), which we also tested experimentally. The inhibitory effects of these molecules have been previously quantified on *E. coli* ADC and inhibition constants have been determined in the mM range [10].

In our recent study [9], 336,761 molecules from three public databases have been targeted: the Maybridge (14,400 molecules), the Zinc (including the National Cancer Institute, 316,181 molecules), and the United States of America Foods and Drug Administration approved drugs database (3,180 molecules). A high-throughput virtual screening, based on the interaction of these compounds with the active site residues of the enzyme by using a stronger binding affinity as compared to that of the MtbADC:fumarate reference (−4.2 kcal/mol) as a positive criterion, led to the identification of 28 lead molecules. Herein, we tested a representative and commercially accessible subset of the positive hits (***I1–I7***, Fig. 1) and compared them under identical experimental conditions to the known ADC inhibitors. Apart from excluding cross-reactivity towards other pyruvoyl-dependent enzymes, all newly tested ligands were selected by ensuring a direct interaction with the well-conserved Arg54, whose role in substrate specificity has already been acknowledged [20], [21].

### Inhibition studies

The enzymatic reactions for inhibitor screening were performed in the presence of 1 mM potential inhibitor, similar to the concentrations employed in previous enzymatic assays [10], [11]. The enzymatic conversion at specific reaction times in the absence and presence of inhibitor was compared and defined as *k*_rel_, which we used as a relative measure of inhibition efficiency (*vide infra*). The results are compiled in Table 1. As expected from literature data [10], mM inhibitor concentrations were required to induce a significant inhibitory effect for most compounds, including the newly identified ones. Since all compounds reported by Williamson and Brown act as competitive inhibitors against ADC [10], it did not come as a surprise that the most effective ones were those containing two carboxylate moieties (***K1–K5***), which are considered to be structural analogues to the natural substrate L-aspartate. In fact, β-hydroxyaspartate (***K2***), *meso*-diaminosuccinate (herein we tested succinate, ***K5***), and cysteic acid (***K4***) have been amongst the first compounds shown to effect the pantothenate pathway [22], [23]. A recent study, where MALDI-TOF mass spectrometry has been employed as a screening methodology for identifying compounds that bind to ADC, has in fact revealed that only a few homologs of L-aspartate, such as L-glutamate (***K3***), can penetrate into the active site of the enzyme [11]. The only two dicarboxylate exceptions constitute the two amino acids L- and D-serine (***K6*** and ***K7***), respectively, which have also been reported to inhibit β-alanine synthesis [10], [24]. Particularly, D-serine had been found to be 4.5 times more potent than its L-enantiomer (*K*_i_ = 0.16 *vs.* 0.73 mM, respectively) [10]. In our direct label-free assays, neither ***K6*** nor ***K7*** showed a considerable inhibitory effect towards ADC activity. However, among all known inhibitors, the most surprising effect was exhibited by oxaloacetate ***K1***, in the presence of which no biocatalytic activity was detected at all (*k*_rel_ = 0).

**Table 1:** Relative inhibitory effects of selected known and newly tested compounds against ADC.[a]

Entry	Compound	Conversion %[b]	*k*_rel_ [c]	Classification
Ref.	None	50	1.00	reference
***Reported compounds*** **** [**10**]				
***K1***	oxaloacetate	0[d]	0.00[d]	very strong
***K2***	β-hydroxyaspartate	18	0.36	strong
***K3***	L-glutamate	20	0.40	strong
***K4***	L-cysteate	20	0.40	strong
***K5***	succinate	32	0.64	moderate
***K6***	L-serine	45	0.90	weak
***K7***	D-serine	48	0.96	insignificant
***Newly tested compounds***				
***I1***	D-tartrate	18	0.36	strong
***I2***	L-tartrate	19	0.38	strong
***I3***	2,4-dihydroxypyrimidine-5-carboxylate	27	0.54	moderate
***I4***	D-tagatose	45[e]	0.90[e]	weak
***I5***	(4S)-1,3-thiazolidin-3-ium-4-carboxylate	48	0.96	insignificant
***I6***	α-D-arabinopyranose	48	0.96	insignificant
***I7***	1,2-dihydropyrazolo[3,4-d]pyrimidin-4-one	48	0.96	insignificant

[a] The measurements were performed using 1 mM L-aspartate, 3 µM ADC, and 1 mM compound (potential inhibitor) in D_2_O at 25°C.

[b] The conversion percentage corresponds to the product formed by integration of the ^1^H NMR signals corresponding to substrate and product of the enzymatic reaction after ca. 30 min upon addition of the enzyme. The time was adjusted to correspond to 50% conversion in the *absence* of inhibitor (reference). The absolute values were averaged from at least two independent assays.

[c] The relative inhibitory effect, *k*_rel_, was calculated as the ratio of the conversion percentages in the presence and absence of compound.

[d] While full inhibition was also observed when using double the enzyme concentration, i.e., 6 µM, only a small nhibitory effect could be detected (*k*_rel_ = 0.9) when the assay was performed with 100 µM oxaloacetate, i.e., at a 10-fold lower inhibitor concentration.

[e] A smaller *k*_rel_ value of 0.74, suggesting moderate inhibition, was observed upon preincubation with ADC for 1 h at ambient temperature.

Complementing the chemoinformatics-based approach, our experimental data show that most molecules possessing a sizable inhibitory potential are, similar to those previously reported [10], anionic in nature. D-tartrate (***I1***, *k*_rel_ = 0.36) was found to be a strong inhibitor, followed by the pyrimidine derivative ***I3*** (*k*_rel_ = 0.54), whose presence reduced the ADC activity by approximately 50%. Presumably, the smaller effect of ***I3***, when compared to ***I1***, is due to a weaker electrostatic binding with the enzyme active site. Surprisingly, D-tagatose (***I4***), which is a neutral compound, exhibited a slight inhibitory effect (*k*_rel_ = 0.74, see footnote in Table 1), but only upon preincubation with the enzyme. It should be mentioned that all compounds in Table 1 were additionally checked under conditions of preincubation with ADC (typically 1 hour, room temperature); however, no differences with respect to the inhibition potential were noted except for D-tagatose (***I4***). The fact that ***I5***, which had emerged as another hit from the chemoinformativs study, showed no detectable inhibitory effect emphasizes the need for complementary computational and experimental studies.

Based on the crystal structure [6], the affinity of molecules to the active site of ADC was postulated to be dominated by two moieties: the pyruvoyl group and the Arg54 residue. All newly tested compounds were selected upon ensuring both a direct interaction with Arg54 residue, while excluding contact with the MtbADC pyruvoyl group [9]. Our data clearly reveal that the inhibitory potential of the tested ligands is, actually, directly dependent on the number of carboxylate groups present. It transpires that in the absence of any ligand interaction with pyruvoyl, Arg54 presides over the active-site binding, which corroborates that this well conserved residue has a fundamental role in substrate specificity, namely by forming a salt bridge with the β-carboxylate of L-aspartate [21].

Apart from compounds ***I1*** and ***I3–I7***, which had been identified as putative inhibitors *via* virtual screening, we have also included L-tartrate (***I2***), the enantiomer of ***I1***, in our experiments. To our surprise, an essentially identical, strong inhibitory effect as that for the D-isomer was observed (*k*_rel_ = 0.38). The D-/L-tartrate example suggests that the chirality of the ligands has no large influence on their binding to the ADC active site, but that the binding is mainly driven by less specific electrostatic interactions.

### Conclusions

In order to counter multi-drug resistance, attenuate *Mycobacterium tuberculosis*'s virulence, and expedite recovery or prolong the survival of patients, additional drugs, in combination with conventional antibiotics, are continuously needed. The development of convenient and easily accessible enzymatic assays combined with drug validation tools are, thus, a compulsory requirement. In this work, we were able to monitor β-alanine formation from L-aspartate during enzymatic conversion *via* ^1^H NMR. This is the first assay reported for ADC, which allows one to accurately monitor the related biocatalytic reaction in a direct and label-free fashion. The functionality of the assay was demonstrated by its use in experimental screening for ADC inhibitors, which allowed the validation of three computationally identified inhibitors against the *Mycobacterium tuberculosis* L-aspartate α-decarboxylase enzyme.
